# Detection of gestational and congenital syphilis in Paraná state, Brazil, 2007-2021: a time series analysis

**DOI:** 10.1590/S2237-96222024V33E2024188.en

**Published:** 2024-06-10

**Authors:** Giovana Gomes de Oliveira, Isadora Gabriella Silva Palmieri, Lucas Vinícius de Lima, Gabriel Pavinati, Vitória Maytana Alves dos Santos, Kelly Cristina Suzue Iamaguchi Luz, Gabriela Tavares Magnabosco

**Affiliations:** 1 Universidade Estadual de Maringá, Departamento de Enfermagem, Maringá, PR, Brazil; 2 Universidade Estadual de Maringá, Programa de Pós-Graduação em Enfermagem, Maringá, PR, Brazil

**Keywords:** Syphilis, Pregnancy, Mother-to-Child Transmission of Infectious Diseases, Syphilis, Congenital, Time Series Studies, Public Health Surveillance, Sífilis, Embarazo, Transmisión Vertical de Enfermedad Infecciosa, Sífilis Congénita, Estudios de Series Temporales, Vigilancia en Salud Pública, Sífilis, Gravidez, Transmissão Vertical de Doenças Infecciosas, Sífilis Congênita, Estudos de Séries Temporais, Vigilância em Saúde Pública

## Abstract

**Objective:**

To describe temporal trends in the detection rates of gestational and congenital syphilis, by maternal age and health macro-region of the state of Paraná, Brazil, 2007-2021.

**Methods:**

This was a time-series study using surveillance data; the trend analysis was performed by means of joinpoint regression, and average annual percent change (AAPC) and 95% confidence intervals (95%CI) were estimated.

**Results:**

An increase in statewide detection of gestational syphilis (AAPC = 21.7; 95%CI 17.7; 32.8) and congenital syphilis (AAPC = 14.8; 95%CI 13.0; 19.7) was found; an increase was also found in the health macro-regions, with the Northwest (gestational, AAPC = 26.1; 95%CI 23.4; 31.6) and North (congenital, AAPC = 23.8; 95%CI 18.8; 48.9) macro-regions standing out; statewide rising trends were observed for young women [gestational, AAPC = 26.2 (95%CI 22.4; 40.6); congenital, AAPC = 19.4 (95%CI 17.6; 21.8)] and adult women [gestational, AAPC = 21.3 (95%CI 16.9; 31.9); congenital, AAPC = 13.7 (95%CI 11.9; 19.3)].

**Conclusion:**

Maternal and child syphilis detection rates increased in the state, regardless of maternal age and health macro-region.

## Study contributions

**Main results:**

Increasing trends were found for the detection rates of gestational and congenital syphilis in Paraná state and its health macro-regions, including in the analysis stratified by maternal age group; however, there was a decline during the COVID-19 period.

**Implications for services:**

There is a need for strategic and immediate action by the state health services, focusing on expanding access and linkage to care, in order to ensure maternal and child well-being and reverse the rising trends observed.

**Perspectives:**

Prevention and control actions towards the elimination of syphilis are needed to overcome these obstacles, directing efforts towards strengthening health education, early detection and appropriate treatment for pregnant women and their partners.

## Resumen

**Objetivo:**

Describir las tendencias temporales en las tasas de detección de sífilis gestacional y congénita, por grupo de edad materna y macrorregión de salud de Paraná, 2007-2021.

**Métodos:**

Estudio de series temporales utilizando datos de vigilancia; se realizó análisis de tendencia mediante regresión segmentada, estimando cambios porcentuales anuales promedio (CPAP) e intervalos de confianza del 95% (IC_95%_).

**Resultados:**

Se identificaron aumentos en la detección estatal de sífilis gestacional (CPAP = 21,7; IC_95%_ 17,7;32,8) y congénita (CPAP = 14,8; IC_95%_ 13,0;19,7); las macrorregiones mostraron incrementos, destacándose la Noroeste (gestacional, CPAP = 26,1; IC_95%_ 23,4;31,6) y la Norte (congénita, CPAP = 23,8; IC_95%_ 18,8;48,9); las tendencias estatales fueron crecientes para mujeres jóvenes [gestacional, CPAP = 26,2 (IC_95%_ 22,4;40,6); congénita, CPAP = 19,4 (IC_95%_ 17,6;21,8)] y adultas [gestacional, CPAP = 21,3 (IC_95%_ 16,9;31,9); congénita, CPAP = 13,7 (IC_95%_ 11,9;19,3)].

**Conclusión:**

Las tasas de detección de sífilis materno-infantil estuvieron en aumento en el estado, independientemente de la edad materna y la macrorregión de salud.

## Resumo

**Objetivo:**

Descrever as tendências temporais nas taxas de detecção de sífilis gestacional e congênita, por faixa etária materna e macrorregião de saúde do Paraná, Brasil, 2007-2021.

**Métodos:**

Estudo de séries temporais, utilizando-se dados de vigilância; realizou-se análise de tendência por regressão segmentada, sendo estimadas variações percentuais anuais médias (VPAM) e intervalos de confiança de 95% (IC_95%_).

**Resultados:**

Foram identificados acréscimos na detecção estadual de sífilis gestacional (VPAM = 21,7; IC_95%_ 17,7;32,8) e congênita (VPAM = 14,8; IC_95%_ 13,0;19,7); as macrorregiões de saúde registraram incrementos, destacando-se as macrorregiões Noroeste (gestacional, VPAM = 26,1; IC_95%_ 23,4;31,6) e Norte (congênita, VPAM = 23,8; IC_95%_ 18,8;48,9); as tendências estaduais foram crescentes para mulheres jovens [gestacional, VPAM = 26,2 (IC_95%_ 22,4;40,6); congênita, VPAM = 19,4 (IC_95%_ 17,6;21,8)] e mulheres adultas [gestacional, VPAM = 21,3 (IC_95%_ 16,9;31,9); congênita, VPAM = 13,7 (IC_95%_ 11,9;19,3)].

**Conclusão:**

As taxas de detecção de sífilis materno-infantil foram ascendentes no estado, independentemente da idade materna e da macrorregião de saúde.

## INTRODUCTION

Syphilis, a sexually transmitted infection, remains a challenge for public health policies in Brazil due to its gestational and congenital forms, which account for a significant portion of infection cases recorded nationally.^1^ ^),(^ ^2^ For example, the Ministry of Health reported 74,095 cases of syphilis among pregnant women and 27,019 among children in 2021 nationwide; in the state of Paraná, especially, 3,223 cases of the infection among pregnant women and 868 among children, were recorded, in the same period.^3^

Programmatic factors such as late initiation of prenatal care, fewer than six prenatal visits and screening failure during pregnancy, are associated with the incidence of these types of infection.^4^ ^)-(^ ^6^ In addition, maternal sociodemographic and behavioral characteristics, such as inconsistent condom use, low monthly income, history of sexually transmitted infection substance use, and aged 35 years and older, are described as predictors of gestational and congenital syphilis.^4^ ^),(^ ^5^ ^),(^ ^7^ ^),(^ ^8^

In Paraná, the main challenges for the control of maternal and child syphilis are related to women aged 20 to 39 years and with low level of education, whose sexual partners are not treated^9^ - possibly due to weaknesses in prenatal care.^10^ In 2023, the state received the “bronze seal” of practices towards the elimination of mother-to-child transmission of syphilis,^11^ primarily following the implementation of the Paraná’s Mother Network in 2012, aiming at the early detection and linkage of pregnant women to prenatal care.^12^

Taking into consideration that maternal characteristics, such as age, and contextual factors, such as place of residence and healthcare access, are relevant to epidemiology and can be considered in monitoring syphilis indicators - especially when targeting the elimination of mother-to-child transmission - this study aimed to describe temporal trends in the detection rates of gestational and congenital syphilis, by maternal age group and health macro-region of the state of Paraná, Brazil, between 2007 and 2021.

## METHODS

*Study design and ethical aspects*

This time-series study involve the use of aggregated data organized over time, regarding the annual detection rates of gestational and congenital syphilis in the state of Paraná. Given that this study design included aggregate and anonymized data, the research project was exempted from the approval of a Research Ethics Committee, in accordance with the National Health Council, Resolutions No. 466 dated December 12, 2012, and No. 674, dated May 6, 2022.

*Setting*

Paraná is the most populous state in the Southern region of Brazil, with a population of 11,444,380 inhabitants and a high human development index of 0.769 in 2021.^13^ In the context of implementing programs and actions and healthcare service provision, the state of Paraná is organized in a decentralized manner into four health macro-regions: East - subdivided into seven health regions -, West, Northwest and North - these with five regions each.^14^

*Participants and data source*

Gestational and congenital syphilis records for the period from 2007 to 2021 were analyzed, taking into consideration data availability in the Notifiable Health Conditions Information System (*Sistema de Informação de Agravos de Notificação* - SINAN) as of October 20, 2023, accessed through the Brazilian National health System Information Technology Department (*Departamento de Informática do Sistema Único de Saúde* - DATASUS);^15^ data on the population of live births were also used, obtained from the Live Birth Information System (*Sistema de Informações sobre Nascidos Vivos* - SINASC), also via DATASUS.^15^

*Variables and statistical methods*

The annual detection rates of gestational and congenital syphilis were calculated according to recommendations and criteria defined by the Ministry of Health:^3^ the total number of cases in pregnant women and children (numerator), according to the year of diagnosis, was divided by the total number of live births (denominator), in the same location and period; and the result was multiplied by 1,000. Rates were estimated by maternal age group [in years: ≤ 19 (young women); ≥ 20 (adult women)], given potential differences in trends between these groups; the denominator was related to maternal age recorded in SINASC.

Trend analysis was performed using segmented linear regression (joinpoint), with a maximum of two inflection points due to the number of years analyzed.^16^ Detection rates were considered as the dependent variable, and years of the series as the independent variable. The grid selection method was applied, transforming the dependent variable into the natural logarithm and adjusting the models to the standard errors of the rates and first-order autocorrelation, assessed according to the data.^16^

In the analysis of the series, we calculated the annual percent change (APC), which provided the increasing (positive) or decreasing (negative) trends at each joinpoint, the average annual percent change (AAPC), which consisted of the geometric mean of the APC, and 95% confidence intervals (95%CI), which indicated the significance of the APC or AAPC when they were different from zero.^16^ The analyses were performed using the Joinpoint Regression Program^®^ (version 5.0.2).

## RESULTS

**Figure 1:**
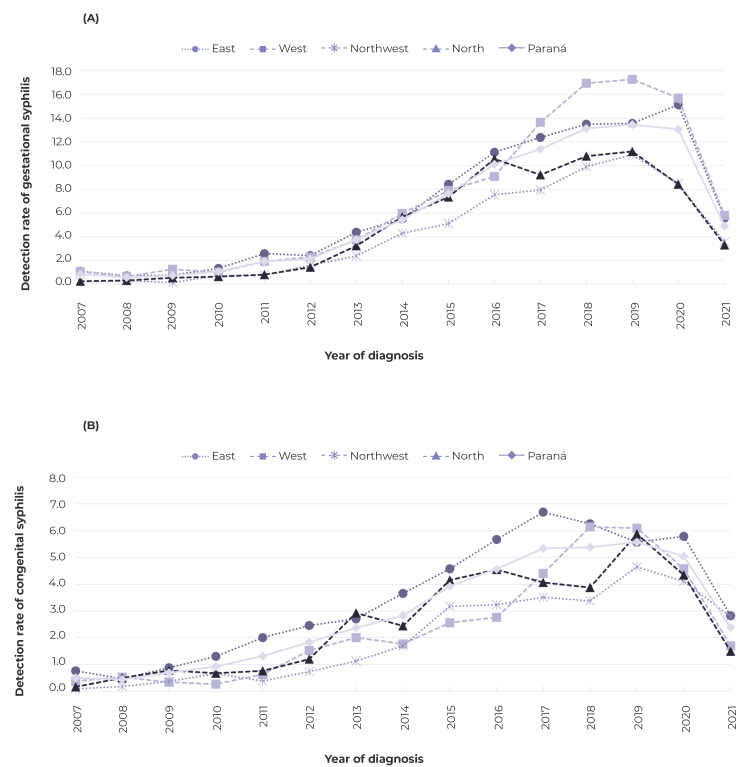
Detection rates of (A) gestational syphilis and (B) congenital syphilis per 1,000 live births, according to health macro-regions, Paraná state, Brazil, 2007-2021

Between 2007 and 2021, a total of 13,861 cases of gestational syphilis and 6,643 cases of congenital syphilis were reported in the state of Paraná, representing detection rates, during the period, of 6.0/1,000 live births and 2.9/1,000 live births, respectively. The East (6.6/1,000 live births) and West (6.9/1,000 live births) health macro-regions showed higher average detection rates of syphilis in pregnant women than the state as a whole (Figure 1A); for congenital syphilis, only the East health macro-region (3.4/1,000 live births) showed a higher average rate than Paraná (Figure 1B).

Increasing trends were found for the detection of gestational syphilis (AAPC = 21.7; 95%CI 17.7; 32.8) and congenital syphilis (AAPC = 14.8; 95%CI 13.0; 19.7) in Paraná state, between 2007 and 2021. The health macro-regions of the state, taken individually, also recorded an increase, with the Northwest standing out, showing an annual increase of 26.1% (95%CI 23.4; 31.6) in gestational syphilis and 24.1% (95%CI 18.2; 56.1) in congenital syphilis; and the North, with an annual increase of 24.2% (95%CI 19.3; 39.5) in gestational syphilis and 23.8% (95%CI 18.8; 48.9) in congenital syphilis (Table 1).

**Table 1:** Temporal trend of the detection rates of gestational syphilis and congenital syphilis per 1,000 live births, according to health macro-regions, Paraná state, Brazil, 2007-2021

	Location	Period	APC^a^ (95%CI^b^)	AAPC^c^ (95%CI^b^)
Gestational syphilis	Paraná	2007-2016	41.4 (30.7;112.3)	21.7 (17.7;32.8)
		2016-2019	13.3 (5.7;43.0)	
		2019-2021	-30.9 (-44.2;-11.5)	
	East	2007-2016	37.7 (13.8;81.7)	20.5 (16.9;27.4)
		2016-2019	11.5 (-5.1;47.7)	
		2019-2021	-25.9 (-41.2;-3.5)	
	West	2007-2019	33.4 (30.9;44.4)	18.7 (14.6;26.6)
		2019-2021	-41.1 (-58.8;-16.7)	
	Northwest	2007-2015	55.2 (48.8;72.3)	26.1 (23.4;31.6)
		2015-2019	19.2 (12.5;25.6)	
		2019-2021	-38.6 (-45.7;-32.1)	
	North	2007-2016	54.9 (45.2;117.6)	24.2 (19.3;39.5)
		2016-2019	2.2 (-7.7;55.1)	
		2019-2021	-38.4 (-53.0;-14.5)	
Congenital syphilis	Paraná	2007-2015	32.8 (29.1;48.0)	14.8 (13.0;19.7)
		2015-2019	11.5 (6.2;19.8)	
		2019-2021	-32.4 (-40.4;-20.0)	
	East	2007-2017	27.2 (23.6;33.5)	13.9 (11.2;17.3)
		2017-2021	-13.6 (-24.8;-5.1)	
	West	2007-2019	28.2 (23.8;44.7)	13.3 (6.8;24.2)
		2019-2021	-46.1 (-68.8;-14.6)	
	Northwest	2007-2015	44.4 (-3.3;595.4)	24.1 (18.2;56.1)
		2015-2019	13.5 (6.0;83.5)	
		2019-2021	-18.8 (-36.9;3.5)	
	North	2007-2015	44.1 (2.2;381.1)	23.8 (18.8;48.9)
		2015-2019	12.5 (4.9;85.5)	
		2019-2021	-18.2 (-34.1;2.6)	

a) APC: Annual percent change; b) 95%CI: 95% confidence interval (lower limit; upper limit); c) AAPC: Average annual percent change.

Among young women, during the same period, increasing trends were found in the detection of gestational syphilis (AAPC = 26.2; 95%CI 22.4; 40.6) and congenital syphilis (AAPC = 19.4; 95%CI 17.6; 21.8) in the state as a whole; Moreover, all health macro-regions recorded an increase in the detection of gestational and congenital syphilis in the age group up to 19 years. Among adult women aged 20 years and older, similarly increasing trends were observed for gestational syphilis (AAPC = 21.3; 95%CI 16.9; 31.9) and congenital (AAPC = 13.7; 95%CI 11.9; 19.3) in Paraná; also in this age group, the health macro-regions followed the statewide pattern (Table 2).

**Table 2:** Temporal trend in the detection rates of gestational syphilis and congenital syphilis per 1,000 live births by maternal age group, according to health macro-regions, Paraná state, Brazil, 2007-2021

	Location	Period	APC^a^ (95%CI^b^)	AAPC^c^ (95%CI^b^)
Gestational syphilis (≤ 19 years old)	Paraná	2007-2016	49.6 (44.5;155.2)	26.2 (22.4;40.6)
		2016-2019	19.1 (11.5;44.6)	
		2019-2021	-36.0 (-52.1;-16.0)	
	East	2007-2018	41.6 (37.8;50.9)	24.6 (21.4;30.4)
		2018-2021	-22.1 (-32.5;-7.1)	
	West	2007-2019	43.2 (40.4;58.9)	25.3 (21.1;36.3)
		2019-2021	-43.7 (-62.2;-16.9)	
	Northwest	2007-2016	53.0 (49.9;65.7)	26.6 (23.9;31.6)
		2016-2019	15.4 (9.1;32.8)	
		2019-2021	-38.2 (-48.4;-24.4)	
	North	2007-2014	76.5 (56.4;532.2)	28.5 (20.0;62.7)
		2014-2019	18.6 (7.6;36.8)	
		2019-2021	-48.2 (-70.3;-20.3)	
Gestational syphilis (≥ 20 years old)	Paraná	2007-2016	39.7 (18.0;113.3)	21.3 (16.9;31.9)
		2016-2019	13.3 (5.8;45.7)	
		2019-2021	-28.7 (-42.7;-8.2)	
	East	2007-2017	34.4 (29.1;47.6)	21.7 (16.8;28.7)
		2017-2021	-5.0 (-27.2;7.3)	
	West	2007-2019	30.9 (28.6;38.8)	17.1 (13.8;22.6)
		2019-2021	-39.9 (-54.3;-18.7)	
	Northwest	2007-2014	65.2 (55.7;87.4)	29.1 (26.1;34.7)
		2014-2019	23.2 (18.4;27.8)	
		2019-2021	-38.7 (-45.1;-32.7)	
	North	2007-2015	66.4 (54.2;120.9)	28.5 (23.2;43.8)
		2015-2019	9.7 (1.5;33.1)	
		2019-2021	-37,4 (-53,5;-16,5)	
Congenital syphilis (≤ 19 years old)	Paraná	2007-2014	41.0 (37.3;49.0)	19.4 (17.6;21.8)
		2014-2019	20.8 (16.6;24.9)	
		2019-2021	-35.4 (-43.1;-28.8)	
	East	2007-2011	61.7 (35.8;291.7)	23.6 (16.1;37.6)
		2011-2018	23.7 (14.6;35.7)	
		2018-2021	-13.7 (-51.0;2.1)	
	West	2007-2019	36.3 (32.4;50.0)	17.8 (10.5;28.4)
		2019-2021	-50.9 (-72.0;-16.2)	
	Northwest	2007-2019	34.9 (31.1;48.2)	19.3 (13.9;29.0)
		2019-2021	-42.9 (-62.3;-13.2)	
	North	2007-2019	24.3 (19.4;56.4)	10.5 (2.1;28.4)
		2019-2021	-45.5 (-73.2;9.4)	
Congenital syphilis (≥ 20 years old)	Paraná	2007-2016	29.9 (27.1;50.0)	13.7 (11.9;19.3)
		2016-2019	5.3 (-0.1;24.2)	
		2019-2021	-29.8 (-38.7;-18.6)	
	East	2007-2017	26.2 (23.6;30.8)	13.0 (11.1;15.5)
		2017-2021	-14.3 (-20.1;-7.7)	
	West	2007-2019	27.3 (24.1;39.4)	14.4 (8.5;23.1)
		2019-2021	-39.7 (-61.8;-12.4)	
	Northwest	2007-2016	42.4 (33.9;67.6)	26.0 (21.4;36.0)
		2016-2021	1.1 (-12.5;10.7)	
	North	2007-2015	36.0 (2.3;443.7)	14.0 (8.3;37.8)
		2015-2019	9.0 (-0.7;66.2)	
		2019-2021	-38.4 (-60.3;-6.1)	

a) APC: Annual percent change; b) 95%CI: 95% confidence interval (lower limit; upper limit); c) AAPC: Average annual percent change.

## DISCUSSION

A pattern of increasing detection rates of gestational and congenital syphilis was observed in the state of Paraná in the period prior to 2019, regardless of the health macro-region and maternal age group. There was also a significant downward trend in almost all macro-regions evaluated during the years of the COVID-19 pandemic, possibly due to weaknesses related to detection and reporting in that emergency scenario.^17^

Present study has limitations inherent to secondary data, which are usually subject to underreporting, incompleteness, inconsistency and under-detection. This occurs mainly due to the possibility that any greater or lower detection or number of notifications, entrusted to health professionals, may lead to a significant or attenuated increase in rate trends. Strategies aimed at improving the quality and completeness of records may benefit future research and evidence-based decision-making.

Time series studies, using data from the SINAN, have highlighted increasing trends in maternal and child syphilis. In the state of Goiás, from 2007 to 2017, there was an increase in the occurrence among pregnant women (APC = 18.0; 95%CI 15.3; 20.8), as well as in congenital syphilis rates (APC = 16.8; 95%CI 20.1; 33.8).^18^ Similarly, in Minas Gerais, an increasing trend was observed for detecting cases during pregnancy (APC = 36.7; 95%CI 32.5; 41.0) and congenital syphilis cases (APC = 32.8; 95%CI 28.0; 37.8), between 2009 and 2019.^19^

Several factors may account for the positive variations in syphilis in pregnant women, including: improvement in surveillance systems and services, contributing to case notification and registration;^20^ ^)-(^ ^22^ expanding the supply for rapid tests, enhancing access to diagnosis;^22^ strengthening prenatal care actions in the state through the implementation of the Paraná’s Mother Network;^12^ and increased socioeconomic inequalities, making women who experience worse conditions more susceptible.^20^ ^)-(^ ^22^

Without disregarding the improvement of public policies for maternal and child care in Paraná,^23^ aimed at the elimination of mother-to-child transmission - and recognized with the achievement of the “bronze seal” - the findings of this study raise an alert to the increasing incidence of congenital syphilis. This situation may be due to, among other factors, screening failures during prenatal visits,^24^ ^)-(^ ^26^ delayed diagnosis of maternal infection^24^ ^)-(^ ^26^ and inadequate treatment management, either in the pregnant woman or her partner.^24^ ^)-(^ ^26^

The predominance of gestational and congenital syphilis in the East and West health macro-regions of Paraná has already been reported.^9^ ^),(^ ^27^ East macro-region encompasses Curitiba, the capital of the state of Paraná, and its metropolitan region, which is more densely populated, while West macro-region is the border region between the state of Paraná, Brazil, Argentina and Paraguay. This factor should be taken into account when interpreting the calculated rates, since lower socioeconomic status has been associated with a higher likelihood of getting syphilis in the state.^27^

It can be concluded that the state of Paraná showed an increase in the detection rates of gestational and congenital syphilis, regardless of maternal age. This increase was more significant in the Northwest and North health macro-regions of the state. However, there was a decline during the COVID-19 pandemic. These findings highlight the need for strengthening health education actions,^28^ expanding testing and treatment for pregnant women and their partners,^29^ and improving access to and close relationship to effective maternal and child care.^30^
